# Genome-wide identification of the *B3* gene family in soybean and the response to melatonin under cold stress

**DOI:** 10.3389/fpls.2022.1091907

**Published:** 2023-01-13

**Authors:** Chunyuan Ren, Huamei Wang, Zhiheng Zhou, Jingrui Jia, Qi Zhang, Changzhi Liang, Wanting Li, Yuxian Zhang, Gaobo Yu

**Affiliations:** ^1^ College of Agriculture, Heilongjiang Bayi Agricultural University, Daqing, Heilongjiang, China; ^2^ College of Horticulture and Landscape Architecture, Heilongjiang Bayi Agricultural University, Daqing, Heilongjiang, China

**Keywords:** *B3* gene family, melatonin, cold stress, soybean, gene expression

## Abstract

**Introduction:**

Melatonin is a multipotent molecule that exists widely in animals and plants and plays an active regulatory role in abiotic stresses. The B3 superfamily is a ubiquitous transcription factor with a B3 functional domain in plants, which can respond temporally to abiotic stresses by activating defense compounds and plant hormones. Despite the fact that the *B3* genes have been studied in a variety of plants, their role in soybean is still unknown.

**Methods:**

The regulation of melatonin on cold resistance of soybean and the response of *B3* genes to cold stress were investigated by measuring biochemical indexes of soybean. Meanwhile, the genome-wide identification of *B3* gene family was conducted in soybean, and *B3* genes were analyzed based on phylogeny, motifs, gene structure, collinearity, and cis-regulatory elements analysis.

**Results:**

We found that cold stress-induced oxidative stress in soybean by producing excessive reactive oxygen species. However, exogenous melatonin treatment could increase the content of endogenous melatonin and other hormones, including IAA and ABA, and enhance the antioxidative system, such as POD activity, CAT activity, and GSH/GSSG, to scavenge ROS. Furthermore, the present study first revealed that melatonin could alleviate the response of soybean to cold stress by inducing the expression of *B3* genes. In addition, we first identified 145 *B3* genes in soybean that were unevenly distributed on 20 chromosomes. The *B3* gene family was divided into 4 subgroups based on the phylogeny tree constructed with protein sequence and a variety of plant hormones and stress response cis-elements were discovered in the promoter region of the *B3* genes, indicating that the *B3* genes were involved in several aspects of the soybean stress response. Transcriptome analysis and results of qRT-PCR revealed that most GmB3 genes could be induced by cold, the expression of which was also regulated by melatonin. We also found that *B3* genes responded to cold stress in plants by interacting with other transcription factors.

**Discussion:**

We found that melatonin regulates the response of soybean to cold stress by regulating the expression of the transcription factor *B3* gene, and we identified 145 *B3* genes in soybean. These findings further elucidate the potential role of the *B3* gene family in soybean to resist low-temperature stress and provide valuable information for soybean functional genomics study.

## 1 Introduction

Soybean is one of the most important crops in the world and are widely grown around the world. However, soybean growth is threatened by different abiotic stresses including salinity, drought, and extreme temperature (Zhang et al., 2020). Cold is an important environmental factor that limits plant growth and reduces crop productivity and quality (Zheng et al., 2021). Under the condition of low temperatures, plants exhibit a variety of cold-induced physiological and biochemical reactions, including the production of reactive oxygen species, changes in membrane lipid composition and the osmotic fluid, and so on (Yuan et al., 2018). Therefore, it is very important to explore the mechanism of cold resistance of soybean to improve the yield and quality.

Transcription factors are considered one of the most important regulators of plant gene expression, which play an important role in various abiotic stress resistance. Hou et al. (2020) found that inhibiting the expression of *NAC* genes would increase the sensitivity of pepper to cold stress. *bZIP73* transcription factor has been revealed to improve the low-temperature stress tolerance of rice seedlings (Liu et al., 2018; Liu et al., 2019). In addition, the B3 transcription factor plays an important role in the cold stress of rubber trees (Gong et al., 2018). The B3 superfamily is a ubiquitous transcription factor with a B3 functional domain (a highly conserved domain that binds to DNA) in plants, and it is also one of the unique transcription factors in plants (Peng and Weselake, 2013). The B3 superfamily consists of several different gene families, including ARF (Auxin response factor), LAV (Leafy cotyledon2 (LEC2) – Abscisic acid insensitive3 (ABI3)–VAL), REM (Reproductive meristem) and RAV (Related to ABI3 and VP1) families (Swaminathan et al., 2008). It has been shown that different ARFs regulated the content of soluble sugars, promoted root development, and maintained chlorophyll content to be resistant to drought and salt stress (Verma et al., 2022). The results of Verma and Bhatia (2019) also indicated that the B3 superfamily of chickpeas could respond to abiotic stresses.

Melatonin is a multipotent molecule that exists widely in animals and plants (Zhan et al., 2019). Melatonin plays a positive role in the regulation of plants in response to abiotic stresses including drought, low temperature, and saline-alkali stress (Arnao and Hernández-Ruiz, 2021). In recent years, stress resistance has been verified to be improved by the over-expression of genes coding melatonin synthesis in *Arabidopsis thaliana*, rice, tomato, and other plants (Park et al., 2013; Zuo et al., 2014; Wang et al., 2014). However, the regulation of melatonin on B3 transcription factors under abiotic stresses has not been reported. Therefore, we first explored the response of exogenous melatonin on soybean to cold stress by regulating B3 transcription factors. At the same time, the whole genome identification of the soybean B3 superfamily was characterized. And the potential function of the B3 superfamily in the abiotic stress response of soybean was further clarified by analysis of the phylogenetic relationship, chromosomal location, expression pattern, and structure of the protein.

## 2 Materials and methods

### 2.1 Plant materials and treatments

The variety Nannong 513 provided by Nanjing Agricultural University, was a temperature-sensitive variety. The seeds were grown in pots with peat soil and vermiculite (7:3), the germination was conducted in a plant incubator, and the temperature was set at 25°C. Four treatment groups were set in this experiment with three independent biological replicates. The plants were treated at the V1 stage of soybean (the first trefoil stage in the vegetative growth period of soybean), and the treated plants were pretreated with foliar application of exogenous melatonin (100 μmol/L) at night, while the other plants were sprayed with distilled water as control. After two days of spraying, half of the plants in both groups were put in another plant incubator at 4°C to conduct cold stress, while the other plants were still kept at 25°C as normal temperature control (Gai et al., 2020). Plant leaves were sampled at 24 h after cold treatment for transcriptome analysis and qRT-PCR detection, and 3 d after cold stress for physiological and biochemical determination.

### 2.2 Physiological response analysis of soybean to cold stress and melatonin treatment

The determination of the activity of peroxidase (POD) and catalase (CAT), and the glutathione redox homeostasis (GSH/GSSG), hydrogen peroxide (H_2_O_2_), and malondialdehyde (MDA) were conducted with POD, CAT, GSH, GSSG, H_2_O_2_, and MDA assay kits, respectively (Suzhou Grace Biotechnology Co., Ltd.). Evans Blue staining was performed according to the method of Xia et al. (2009).

The melatonin (MT) content in soybean was determined according to Yan et al., 2019, leaves were ground to a powder in liquid nitrogen and then extracted with 1.5 mL of chloroform at 4°C for 15 h. After centrifugation of the extraction mixture, the chloroform fraction was evaporated to dryness and dissolved in 100 µL of 42% methanol. Aliquots of 10 µL were subjected to HPLC using a fluorescence detector system. The contents of abscisic acid (ABA) and indole-3-acetic acid (IAA) were determined by ELISA reagent kits (Shanghai Enzyme-linked Biotechnology Co., Ltd. Shanghai, China) (Yu et al., 2022). The data were subjected to analysis of variance with SPSS, and the means were compared using Duncan’s t-test at the 5% level. meanwhile, the data was visualized using origin 8.0 (microcal Inc, Northampton, mA, USA).

### 2.3 Identification of the *GmB3* genes

Input 118 known *A. thaliana B3* gene IDs into the *Arabidopsis* genome database (TAIR, http://www.arabidopsis.org/, 2020) to obtain protein sequences. Candidates from the soybean B3 family were investigated with BLASTP using *Arabidopsis thaliana* B3 protein sequence as probe. In the SMART database (http://smart.embl-heidelberg.de/, 2020), the domain of Pfam (PF02362) was identified and screened in the phytozome database. Finally, 145 *GmB3* genes were identified and named *GmB3-1*–Gm*B3-145* according to their chromosomal positions. The theoretical isoelectric points (pI), the number of amino acids, and the grand average of hydropathicity (GRAVY) of all predicted B3s was then determined by ExPASy (Wilkins et al., 1999). Multisequence matching and Maximum-Likelihood analysis were performed using 1000 replicates as bootstrap values (Morgulis et al., 2008).

### 2.4 The motif and gene structure analysis of *GmB3* genes

The MEME tool (http://meme.nbcr.net/meme/, 2020) was used to detect the B3s’ motifs (Bailey et al., 2009). The exon-intron sequence of *B3* genes was determined by comparing the coding sequence and genome sequence of *B3* genes using the Gene Structure Display Server (GSDSv2.0; http://gsds.cbi.pku.edu.cn/, 2020) (Hu et al., 2015).

### 2.5 Collinearity analysis

Coordinate correspondence between DNA and protein sequences is determined using Gene-wise (Simmons et al., 2019). Whole-genome protein sequences and gene positions for soybean were retrieved from EnsemblPlants (http://plants.ensembl.org/index.html, 2020), MCScanX was utilized to analyze *B3s* gene duplication events (Wang et al., 2012). TBtools were adopted to visualize the results.

### 2.6 Identification of plant growth regulator-related cis-elements

The upstream 1.5 kilobases (kb) genomic DNA sequences of the GmB3s were retrieved from the soybean genome and the putative cis-regulatory elements in promoter regions were identified using the PlantCare database (http://biinformatics.psb.ugent.be/tools/plantcare/, 2020) (Rombauts et al., 1999).

### 2.7 Expression profiles of *GmB3* genes in diverse tissues

The *B3* gene expression data in different tissues were obtained from the Phytozome database (https://phytozome-next.jgi.doe.gov/pz/, 2022).

### 2.8 Analysis of the expression level of *GmB3* genes from transcriptome data

Plant leaves were sampled at 24 h after cold treatment for transcriptome analysis, each treatment was repeated with three times (Yu et al., 2021). The total RNA was extracted, and the library was built, and the quality was tested, and the transcriptome was sequenced, analyzed and annotated by LC SCIENCES (Hangzhou, China) (Sheng et al., 2017). During the detection of DEGs, a fold-change ≥ 1.5 and a false discovery rate < 0.05 were used to screen different expression genes. Differential expression profiles of differentially expressed genes were presented. Amazing HeatMap software (Chen et al., 2020) was used to generate a heatmap.

### 2.9 RNA extraction and qRT-PCR assays

Total RNA was extracted using TRIzol^®^ reagent (Invitrogen, Carlsbad, CA, USA). One microgram per RNA sample was used as the template for the synthesis of the first-strand cDNA, using the ReverTra Ace™ qPCR RT Master Mix with gDNA Remover (TOYOBO Co., Osaka, Japan). Subsequently, qRT-PCR was performed with SYBR^®^ Select Master Mix RT-PCR System (Takara) on an optical 96-well plate. Select actin as an internal reference. All the primers used for gene expression analysis were shown in **Table S1**. Relative expression level of genes was calculated using formula 2^−ΔΔCT^ (Livak and Schmittgen, 2001; Cui et al., 2019). Three independent biological replicates were analyzed.

### 2.10 Prediction of transcription factors of soybean *B3* gene family

We utilized PlantRegMap (Internet) http://plantregmap.gao-lab.org/index- chinese.php (Tian et al., 2020) to predict transcription factors associated with GmB3. Simultaneously, the transcription factors (TFs) network was visualized using Origin 8.0.

## 3 Result

### 3.1 Effects of exogenous melatonin treatment on oxidative stress in soybean under cold stress

**Figure 1:**
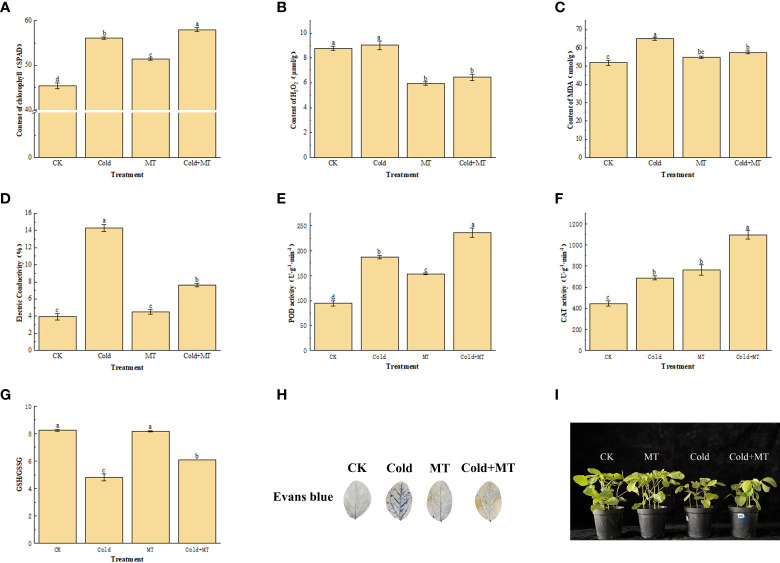
Effects of exogenous melatonin treatment on oxidative stress in soybean. **(A)** Chlorophyll content. **(B)** H_2_O_2_ content. **(C)** MDA content. **(D)** Electrical conductivity. **(E)** POD activity. **(F)** CAT activity. **(G)** GSH content. **(H)** Evans blue staining. **(I)** Phenotype of soybean seedlings. Note: H_2_O_2_: Hydrogen peroxide, MDA: Malondialdehyde, POD: Peroxidase enzyme, CAT: Catalase, GSH: Glutathione. Error bars represented the standard deviation (SD; n = 3). According to Duncan’s multiple tests, bars with different letters were significantly different (p < 0.05).

To assess the cold-caused injury to soybeans, we analyzed its Chlorophyll and H2O2 content, MDA accumulation, and electrical conductivity. The cold treatment significantly increased the content of Chlorophyll, MDA, and electrical conductivity by 23.4%, 29.9%, and 263.6%, respectively, compared with the control. However, exogenous melatonin application substantially decreased the content of MDA and electrical conductivity by 11.4% and 46.7%, respectively, compared with the only cold treatment, and chlorophyll content was further increased. Interestingly, the content of H2O2 in soybean with foliar application of melatonin decreased by 28.8%, compared with cold treatment alone (**Figures 1A–D**). This indicated that exogenous melatonin treatment could alleviate the damage caused by cold stress in soybean.

We measured the activity of antioxidant enzymes including POD and CAT to unveil how melatonin alleviates cold-induced oxidative stress. The cold treatment significantly enhanced the activity of POD and CAT by 97.9% and 54.5%, respectively, compared with the control (**Figures 1E, F**). While compared with cold treatment alone, the activity of POD and CAT increased by 26.4% and 59.3%, respectively, with foliar application of melatonin. In addition, compared with the control, the GSH/GSSG ratio of soybean leaves after cold treatment decreased by 41.6%, compared with the control, but increased by 26.5% after melatonin application, compared with cold treatment alone (**Figure 1G**). The results of Evans blue staining also demonstrated that the damaged tissues in soybean leaves increased after cold treatment, while the application of melatonin alleviated the damage to soybean leaves (**Figure 1H**). Soybean seedling growth was seriously affected by the cold, compared with the control, the development of soybean seedlings was significantly inhibited and the leaf surface was damaged after cold stress, while melatonin treatment alleviated the leaf surface damage of soybean after cold stress (**Figure 1I**). This indicated that exogenous melatonin treatment could improve the antioxidant enzyme activity and GSH/GSSG ratio of soybean against cold stress.

### 3.2 Effect of exogenous melatonin treatment on hormone content in soybeans under cold stress

**Figure 2:**
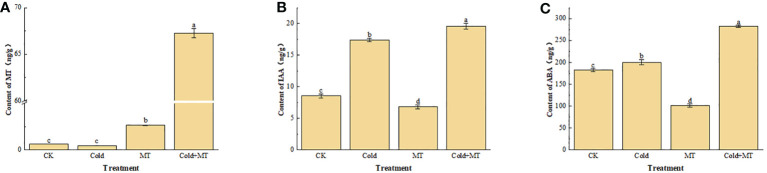
Effects of exogenous melatonin treatment on the content of hormone in soybeans. **(A)** the content of MT. **(B)** the content of IAA. **(C)** the content of ABA. Note: MT: Melatonin, IAA: Indoleacetic acid, ABA: Abscisic acid. Error bars represented the standard deviation (SD; n = 3). According to Duncan’s multiple tests, bars with different letters were significantly different (p < 0.05).

To evaluate the effect of exogenous melatonin on endogenous melatonin and other hormones in soybean under cold stress, the content of melatonin, indoleacetic acid, and abscisic acid in the leaves of soybean seedlings were analyzed under different treatments (**Figure 2**). Compared with the control, the content of endogenous melatonin in soybean seedlings after exogenous melatonin treatment increased in both normal temperature and cold treatment groups, especially for the cold treatment, the melatonin content in soybeans increased more significantly with the application of melatonin. Although no significant difference appeared in melatonin content in soybeans between the cold treatment and control. Interestingly, compared with the control, the cold treatment increased the content of IAA and ABA in soybeans, while both were further increased with the application of melatonin. The results suggested that exogenous melatonin might alleviate soybean response to cold stress by regulating the hormone content in soybeans under cold stress.

### 3.3 Effects of exogenous melatonin treatment on the expression of *B3* genes in soybean under cold stress

**Figure 3:**
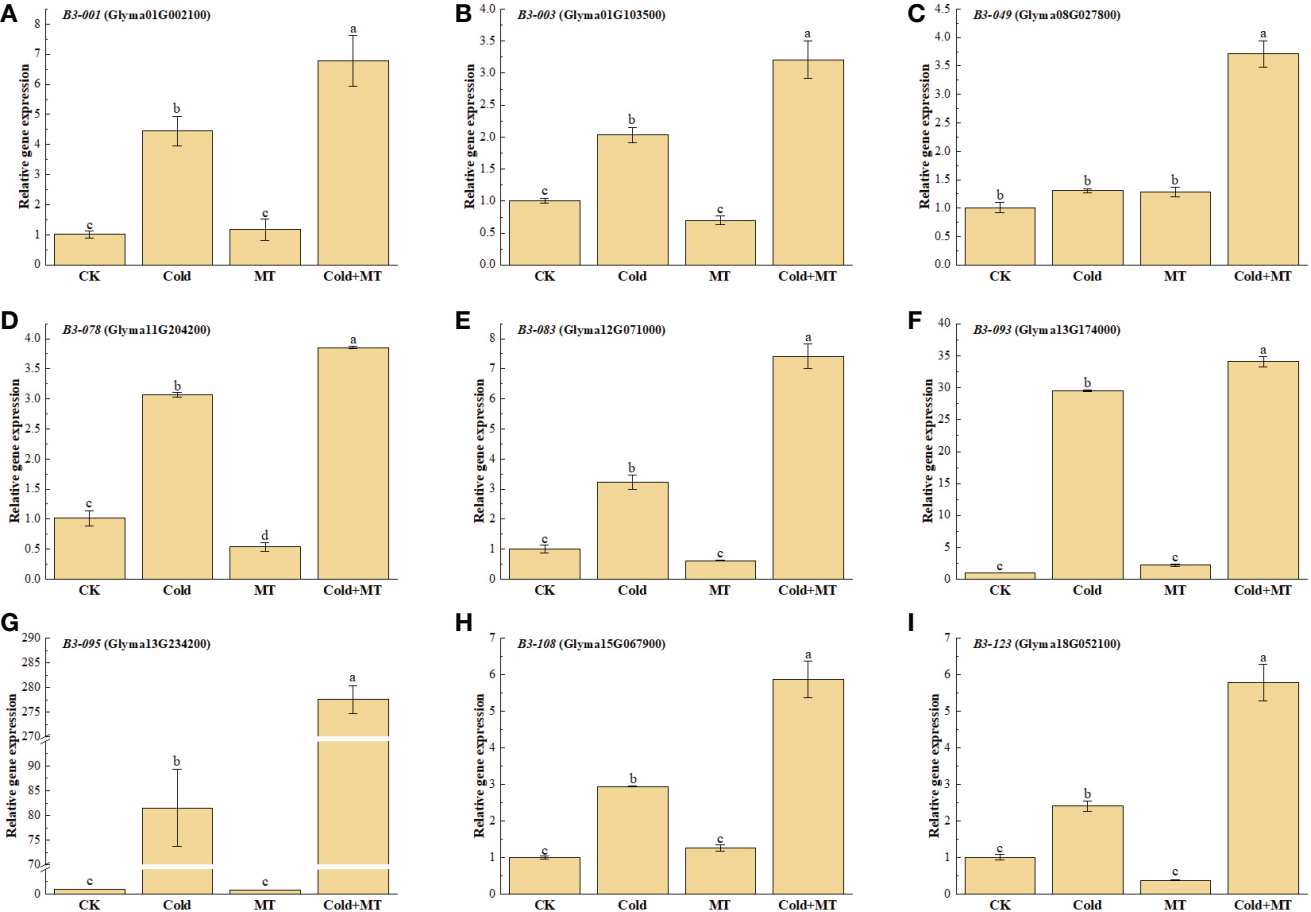
Effects of exogenous melatonin treatment on the expression of *B3* genes in soybean. **(A)** GmB3-001 expression. **(B)** GmB3-003 expression. **(C)** GmB3-049 expression. **(D)** GmB3-078 expression. **(E)** GmB3-083 expression. **(F)** GmB3-093 expression. **(G)** GmB3-095 expression. **(H)** GmB3-108 expression. **(I)** GmB3-123 expression. Error bars represented the standard deviation (SD; n = 3). According to Duncan's multiple tests, bars with different letters were significantly different (p < 0.05).

B3 transcription factors play an important role in plant tolerance to abiotic stress. To further study the effect of exogenous melatonin on cold stress, the expression of several representative soybean *B3* genes was determined after treatment with melatonin under cold stress by qRT-PCR (**Figure 3**). The results explored that the expression of *B3-001*, *B3-003*, *B3-078*, *B3-083*, *B3-093*, *B3-095*, *B3-108*, and *B3-123* was induced by cold treatment and increased by 3.36-fold, 1.03-fold, 2.04-fold, 2.16-fold, 29.52-fold, 80.52-fold, 1.94-fold, and 1.38-fold, respectively, compared with the control. While the application of exogenous melatonin substantially further improved the expression of all the above genes, compared with only cold treatment, which up-regulated by 52.4%, 58.1%, 25.4%, 130.4%, 15.4%, 240.5%, 199.7%, and 141.2%, respectively. Similarly, the transcription of *B3-049* gene was also significantly induced with the application of melatonin, compared with the cold treatment alone, although no significant difference was observed between cold and normal temperature treatment. There was no significant difference in the expression of the above genes between melatonin treatment and control under normal tmperature, except for *B3-078* gene. The above results indicated that melatonin could alleviate the cold stress of soybean by inducing the expression of *B3* genes.

### 3.4 Identification and chromosome distribution of *B3* gene family in soybean

**Figure 4:**
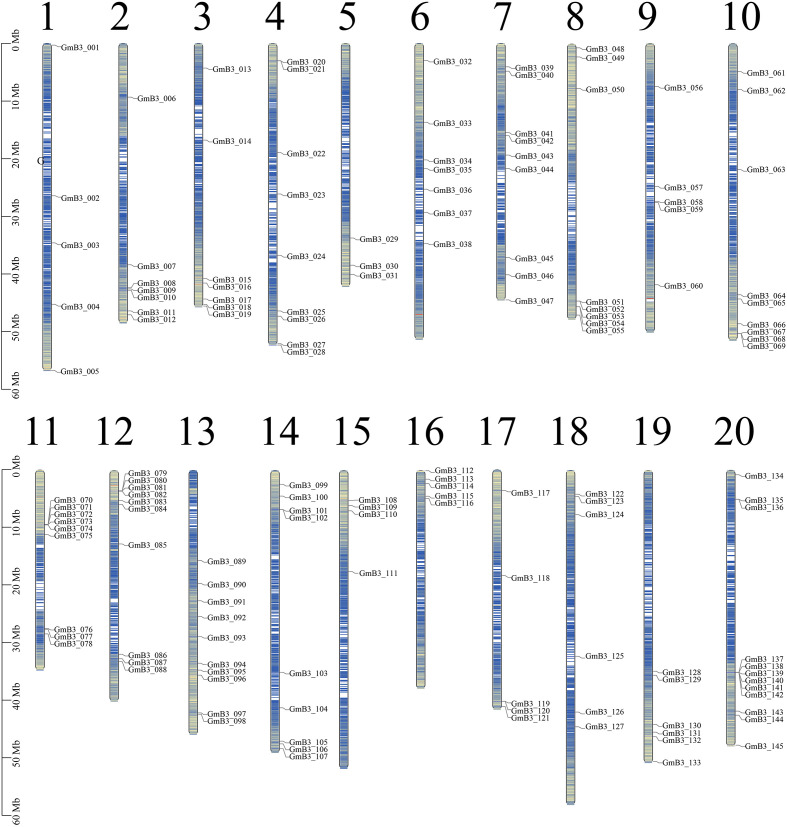
Chromosomal distribution of the 145 *GmB3* genes identified in the present study. The chromosome number was indicated above the chromosome.

We used *Arabidopsis* B3 protein sequence as reference, screened candidate B3 protein through blast comparison of soybean genome, and screened 145 *B3* genes through conservative domain (PF02362) and redundancy elimination. The candidate genes were named *GmB3-001* to *GmB3-145* based on the location on chromosomes (**Figure 4**). The soybean *B3* genes were unevenly distributed on 20 chromosomes, among which the 20th chromosome was focused for the distribution of 12 soybean *B3* genes. The B3 protein sequence consisted of 72-1136 amino acids, with an average length of 538 amino acids. The relative molecular weight and isoelectric point of B3 protein ranged from 8632.16 kDa (*GmB3-140*) to 127058.28 kDa (*GmB3-117*) and 4.86 (*GmB3-100*) to 11.02 (*GmB3-066*), respectively. Among them, 75 B3 proteins were acidic (with an isoelectric point < 7), while 70 B3 proteins were alkaline (isoelectric point > 7) (**Table S2**).

### 3.5 Phylogenetic relationship of *GmB3* genes and synteny analysis of *GmB3* genes

**Figure 5:**
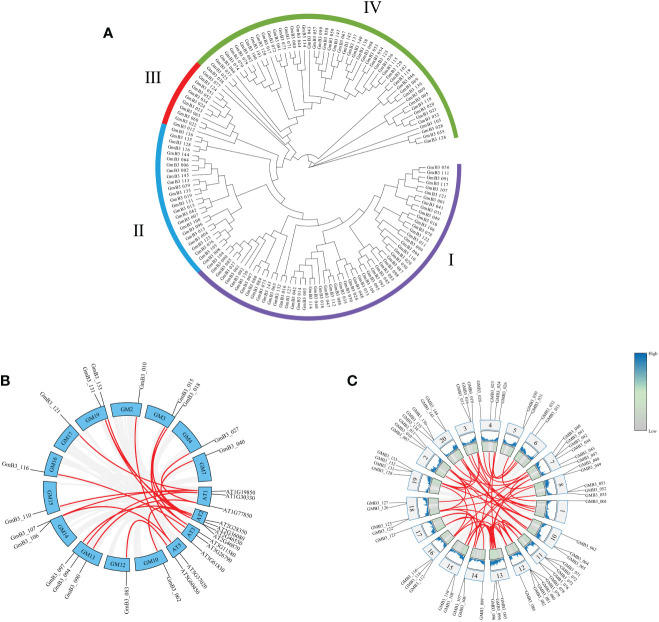
The phylogenetic tree of B3 members and collinear analysis of GmB3s. **(A)** Phylogenetic analysis of *GmB3* genes. **(B)** Collinear map of *B3* genes between soybean and *Arabidopsis*. **(C)** Collinear map of different *GmB3* genes in soybean.

The phylogenetic tree was constructed using the B3 protein sequences of soybean. The B3 family of soybean could be classified into four subgroups (namely B3-I, B3-II, B3-III, and B3-IV) based on the phylogenetic tree analysis. These B3 groups (I-IV) consisted of 57, 25, 11, and 52 members, respectively (**Figure 5A**). To elucidate the evolutionary relationship between the *B3* gene family, we constructed a co-linear map of *B3* genes in *Arabidopsis* and soybean, which revealed that 20 pairs of *B3* genes presented collinearity between soybean and *Arabidopsis* (**Figure 5B**), and 26 pairs of *B3* genes existed collinearity among 14 chromosomes in soybean (**Figure 5C**).

### 3.6 Gene structure, motif pattern, and conserved domain of *GmB3* genes

**Figure 6:**
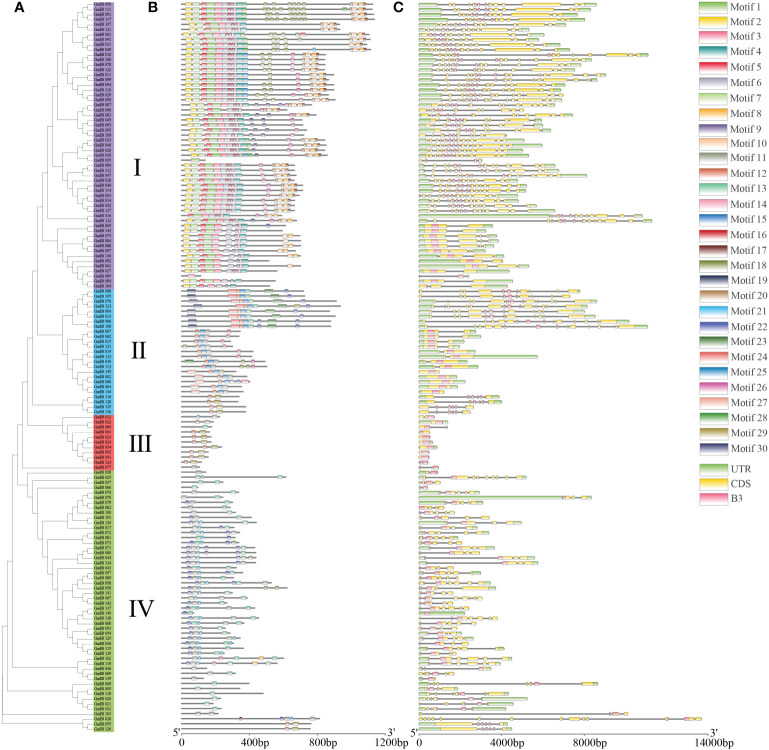
Gene structure and conserved motifs in *GmB3* genes. **(A)** The phylogenetic tree of GmB3s protein. **(B)** The motif composition of GmB3 proteins. Different colors represented different motifs. **(C)** Exon-intron structure and conserved domain of *GmB3* genes.

To identify the conserved structure of soybean B3 protein, 30 motifs were predicted by MEME motif analysis. Similar motif structures were present in the B3 members of the same subgroup, although different motifs appeared in various subgroups. For instance, most B3 proteins of subgroup contained 7 to 25 motifs, except for *GmB3-35* and *GmB3-85*, containing one motif. Most GmB3 proteins of subgroup II contained 2 to 8 motifs, while Motif 30 was only found in subgroups II. Most B3 proteins of subgroup III only contained 2 motifs, although *GmB3-60* and *GmB3-77* contained one motif. And B3 proteins of subgroup IV contained 1 to 7 motifs (**Figures 6A, B**). The B3s structure of exon and intron was detected to obtain gene structure. Although different gene structures were exhibited in four subgroups, a relatively consistent gene structure of B3s was found in each subgroup (**Figure 6C**).

### 3.7 Cis-Elements in the promoter regions of *GmB3* genes

**Figure 7:**
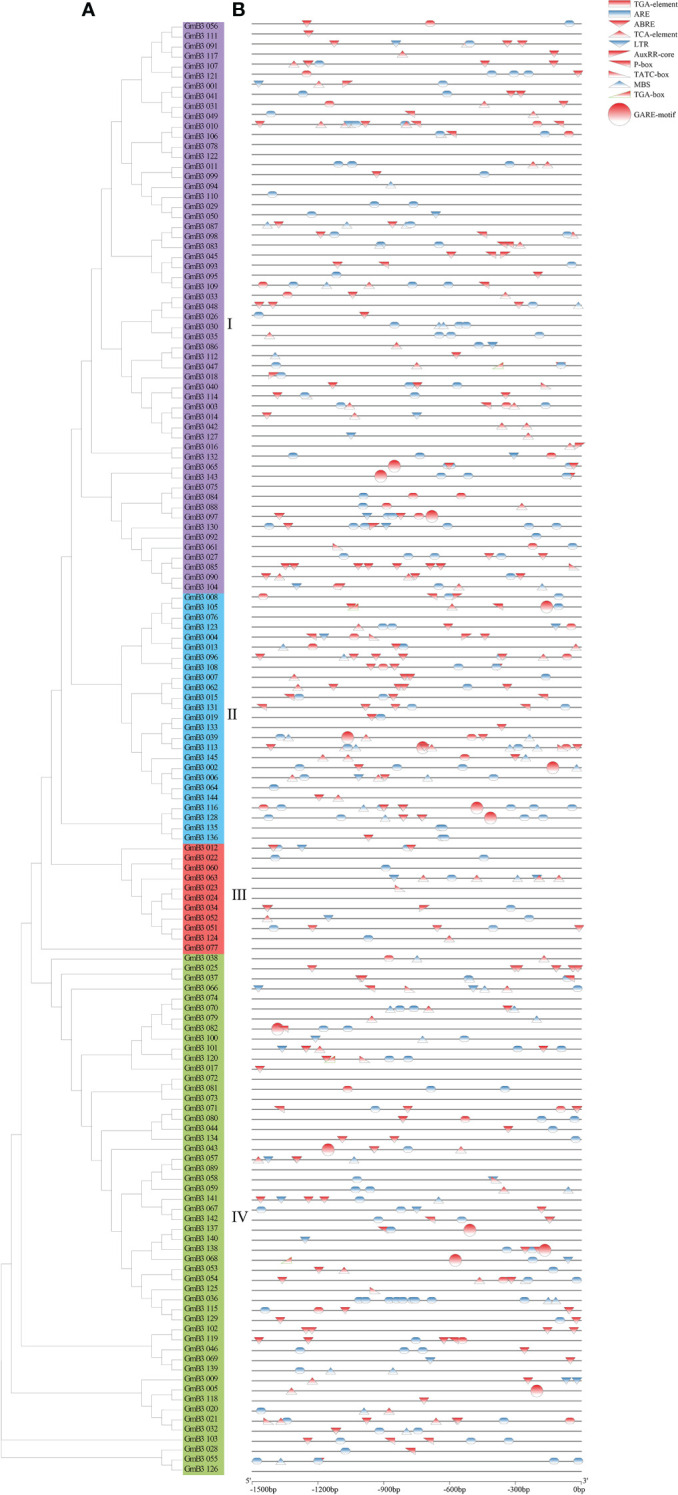
Analysis of the cis-acting elements in GmB3 genes. **(A)** The phylogenetic tree of GmB3s’ protein. **(B)** Cis-elements analysis of GmB3 genes. Different colors represented different cis-acting elements and their position in the GmB3 genes.

To investigate the regulatory mechanism of the *B3* gene, we scanned the sequence of the promoter codon 2000bp upstream of ATG and obtained a number of cis-acting elements associated with plant hormones and stress response. Hormone response elements were mainly induced by auxin (AuxRR-core), gibberellin (GARE), and abscisic acid (ABRE). Among them, 84 *B3* genes were found with ABA response elements, accounting for 57.9% of the total genes. In addition, 7 *B3* genes with auxin response elements and 15 *B3* genes with GA response elements were found. The predicted stress response elements mainly included LTR (low-temperature response), MBS (drought induction), and ARE (anaerobic induction), among which, 103 *B3* genes containing ARE elements that were the most frequent, accounting for 71.0% of the total, while 34 *B3* genes containing MBS and 26 *B3* genes containing LTR were revealed (**Figure 7**). These results suggested that *B3* genes might be involved in the response of soybean to hormones and abiotic stresses.

### 3.8 Expression patterns of GmB3s in different tissues

**Figure 8:**
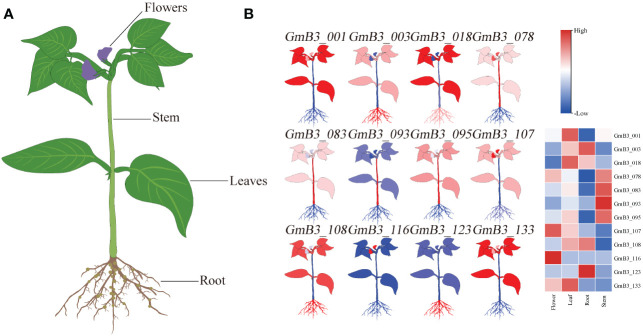
Expression profiles of *GmB3* genes in different tissues. **(A)** The schematic diagram of different tissues of soybean. **(B)** The significant expression of 12 GmB3s in different tissues. The expression level were based on the transcriptome data.

The transcription level of B3 in different tissues (including roots, stems leaves, and flowers) was analyzed based on the Phytozome database. Remarkable difference appeared in B3s expression levels of various soybean tissues (**Figure 8**). Thereinto, the expression of *GmB3-116* was only high in flower, while the expression of *GmB3-093* was only high in stem. However, *GmB3-123* was only highly expressed in root, while *GmB3-078* was only weakly expressed in root. These results suggested that *B3* genes were expressed in a tissue-specific manner in the soybean.

### 3.9 Expression of *GmB3* gene and interactional transcription factors under different treatments

**Figure 9:**
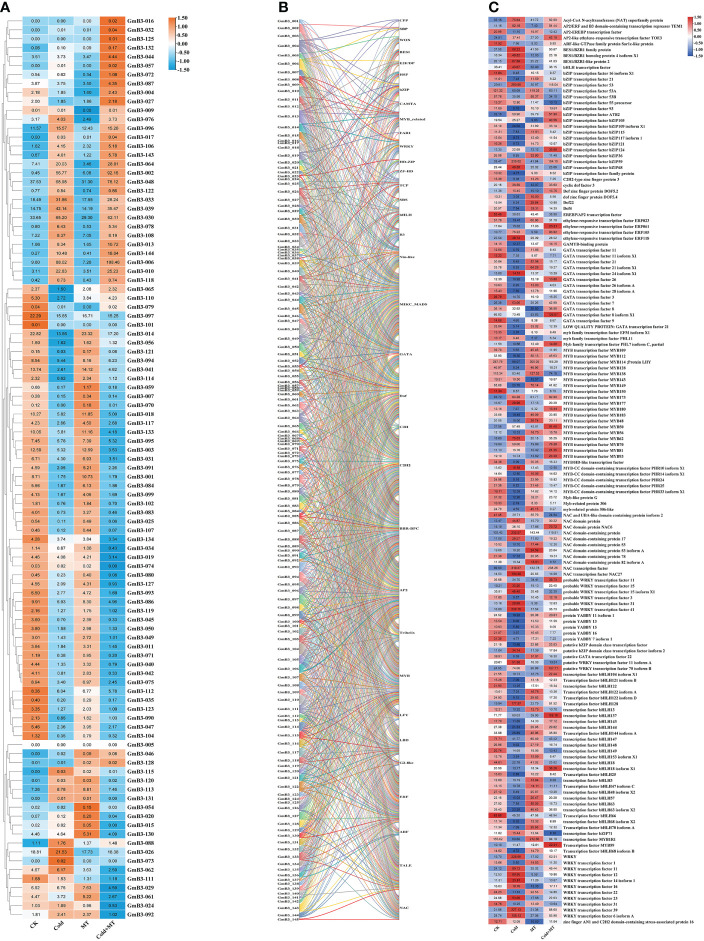
Network analysis of soybean *B3* genes and transcription factors. **(A)** Expression level of *B3* genes under different treatments. **(B)** Relationship between the *B3* gene and other transcription factors. **(C)** Expression level of other transcription factors under different treatments. Different colors represent different GmB3 genes.

Based on the transcriptome data, the response of *B3* gene family members to cold and melatonin was analyzed. Compared to control, 39 *B3* genes were induced and 59 *B3* genes were inhibited by cold stress. The transcription of 40 *B3* genes of soybean was induced, however, the expression of 57 *B3* genes was reduced by melatonin treatment compared with the control. Similarly, the transcription of 42 *B3* genes was enhanced, while the expression of 57 *B3* genes was decreased by cold and melatonin treatment compared to the cold treatment alone (**Figure 9A**). It suggested that *B3* genes responded to melatonin under cold treatment in soybean.

To further investigate the regulatory mechanism of the *B3* gene of soybean, we predicted that a total of 34 transcription factor families interacted with the *B3* gene. Among all *B3* genes, 18 transcription factors were predicted to interact with *GmB3-004* and *GmB3-013*, with the largest number (**Figure 9B**). Interestingly, transcriptome data revealed that 14 transcription factor families contained genes that could be induced by cold, while regulated by melatonin treatment (**Figure 9C**). The results indicated that the *B3* gene regulated soybean stress response through interaction with transcription factors, suggesting that the *B3* gene played an important role in plant resistance to abiotic stress through interaction with transcription factors.

## 4 Discussion

Low temperature is a typical abiotic stress factor which has been reported to limit plant growth and yield (Shen et al., 2022). Plants have evolved various strategies for sensing and responding to cold stress. Nevertheless, how plants perceive cold signals remains an important question to be addressed. Plants may sense and transmit cold signals to cells through a variety of mechanisms, including reactive oxygen species (ROS), calcium (Ca^2+^), and plant hormone signals (Liu et al, 2022). Melatonin is a pleiotropic molecule widely existing in animals and plants, which has a positive regulatory role in drought, low temperature, salinity, and other abiotic stresses (Zhan et al, 2019; Arnao and Hernández-Ruiz, 2021). It has been shown that exogenous melatonin could improve cold stress tolerance in *Arabidopsis*, tomato, cucumber, and other plants (Bajwa et al., 2014; Ding et al., 2017; Zhao et al., 2017). In this study, exogenous melatonin treatment significantly increased the content of endogenous melatonin in soybean compared with control or cold treatment alone. Melatonin is an important modulator of gene expression related to plant hormones, such as auxin carrier proteins and the metabolism of gibberellins, cytokinins, abscisic acid, indole-3-acetic acid, and ethylene. The results showed that melatonin could effectively promote the plant growth and development under stresses (Arnao and Hernández-Ruiz, 2018). Similarly, melatonin treatment alone decreased auxin and abscisic acid contents in soybeans, while melatonin application under cold stress increased auxin and abscisic acid contents in soybeans. This suggests that melatonin could alleviate cold stress in soybean by interacting with other hormones.

Extreme temperature could cause the generation of ROS, such as superoxide, hydroxyl radicals, hydrogen peroxide, and singlet oxygen, to restrict plant growth and development (Bita and Gerats, 2013; Hasanuzzaman et al., 2020). Plants respond to abiotic stress through increasing the content of antioxidant substances and enhancing the activity of antioxidant enzyme to remove harmful ROS and free radicals, to against a series of stresses (Roy et al., 2014). Both GSH and AsA, the important antioxidant substances, and the antioxidant enzymes, including APX, SOD, POD and CAT, contribute to alleviate the oxidative stress damage in plants (Noreen and Ashraf, 2009). It has been revealed that exogenous melatonin could promote the activity of antioxidant enzymes in plants under stress, such as *Arabidopsis*, oilseed rape and tomato, and improve plant resistance (Zeng et al., 2018; Siddiqui et al., 2019). Raza et al. (2022) explored that exogenous melatonin improved plant tolerance against extreme temperature either by directly scavenging ROS molecules or indirectly by improving photosynthetic efficacy, antioxidant enzyme activities, and metabolite contents in plants. Our study also revealed that, compared with cold stress alone, melatonin application significantly reduced the content of H_2_O_2_ and MDA and the electrical conductivity of soybeans, and increased the content of chlorophyll in soybeans. This suggested that exogenous melatonin treatment could remove excessive ROS in soybean, and alleviate membrane lipid peroxidation under cold stress, thereby improving the tolerance of soybean to cold stress.

The B3 superfamily is a ubiquitous transcription factor with a B3 functional domain (a highly conserved domain that binds to DNA) in plants (Peng and Weselake, 2013), which consists of several different gene families, including LAV, ARF, RAV, and REM families (Swaminathan et al., 2008), and the B3 transcription factor plays an important role under abiotic stress in plants (Gong et al., 2018). Kang et al. (2018) found that the over-expressed sweet potato *B3* gene *IbARF5* in transgenic *Arabidopsis thaliana* enhances the resistance to drought and salt stress through carotenoid biosynthesis. In addition, the *B3* gene can also participate in regulating the tolerance of *Arabidopsis thaliana* and rapeseed to cold stress (Yamasaki et al., 2004; Luo et al., 2019). In the present study, the expression of *B3* genes (including *B3-001, B3-003, B3-078, B3-083, B3-093, B3-095, B3-108*, and *B3-123*) increased after cold treatment compared with the control, which was substantially further induced with foliar application of melatonin, compared with the only cold treatment. It revealed that melatonin could improve the tolerance to cold stress in soybean by inducing the expression of *B3* genes.

To further understand the *B3* gene family in soybean, a total of 145 *B3* genes were identified in soybean, which was more than that in *Arabidopsis* (118 *B3* genes), tobacco (114 *B3* genes), and common bean (110 *B3* genes) (Swaminathan et al., 2008; Xia et al., 2019; Du et al., 2022). On the basis of phylogenetic analysis, these *B3* genes were clustered into four groups and all 145 B3 members exhibited typical characteristics of the *B3* gene domain. Introns are important components of genes. Despite the lack of involvement in protein coding, intron acquisition or loss and intron insertion position are widely regarded as key clues to explore the evolutionary diversity of gene families (Rogozin et al., 2000). Gene structure and motif analysis explored that similar gene structures, motifs, and cis-regulatory elements exhibited in each of the *B3* gene subgroups, which supported the reliability of the subfamily classification. These findings were in agreement with previous studies on *Arabidopsis*, tobacco, and common bean.

The soybean *B3* genes were predicted to proceed similar functions with *Arabidopsis* B3 family members because of homologous genes with high collinearity (Gazzarrini et al., 2004). It has been revealed that most of the 20 *B3* genes of *Arabidopsis* that were collinear with soybean were associated with auxin signal response. For instance, *AT1G19850* (collinear with *GmB3-107*), *AT2G46530* (collinear with *GmB3-018*), and *AT3G61830* (collinear with *GmB3-018*) respond to auxin (Varaud et al., 2011; Möller et al., 2017), and *AT2G46870* (collinear with *GmB3-133*) and *AT3G26790* (collinear with *GmB3-116*) are involved in abiotic stress resistance (Chiu et al., 2012; Sato et al., 2018). Taken together, *B3* genes may be involved in soybean response to abiotic stresses and hormones.

Abiotic stress resistance is one of the important characteristics of soybean breed improvement (Wang and Komatsu, 2018). In recent years, the *B3* genes in *Arabidopsis* and rapeseed have been found to be involved in defense against cold stress (Yamasaki et al., 2004; Luo et al., 2019). Here, transcriptome data revealed that most *B3* genes could be induced or inhibited by cold, and their expression changes after melatonin treatment. This led to the suggestion that the *B3* gene family played an important role in plants against cold stress, which may be regulated by melatonin. qRT-PCR analysis further supported the above findings.

Transcription factors are important proteins, which bind to specific DNA motifs, regulate the transcription level of genes, and play a significant role in the stress response of plants (Wu et al., 2022). We have revealed 144 soybean *B3* genes, which could interact with 34 different transcription factors, and most of them were regulated by melatonin under low temperature conditions. Thus, melatonin can relieve cold stress in soybeans by regulating the interaction of the *B3* gene with transcription factors. In addition, among them, 91 *B3* genes had complex associations with C2H2 zinc finger proteins transcription factors, 87 *B3* genes had complex associations with MYB transcription factors, and 86 *B3* genes had complex associations with AP2 transcription factors, and the C2H2, MYB and AP2 transcription factor are associated with plant abiotic stress (Feng et al., 2020; Han et al., 2020; Dar et al., 2022). Therefore, *B3* genes can be involved in regulating stress through its interaction with these transcription factors, but the mechanism is need to be further studied.

## 5 Conclusion

In summary, we found that cold stress induced oxidative stress in soybean by producing excessive reactive oxygen species. However, exogenous melatonin treatment could enhance the antioxidative system, including POD activity, CAT activity, and GSH/GSSG, to scavenge ROS. Furthermore, exogenous melatonin treatment could increase the content of endogenous melatonin and other hormones, such as IAA and ABA, and induce the expression of *B3* genes to alleviate cold stress in soybean. In addition, 145 *GmB3* genes were identified from the soybean genome. The B3s members were divided into four subgroups based on the analysis of the composition, phylogenetics, motifs, gene structure, collinearity, and cis-regulatory elements. Interestingly, transcriptome data and qRT-PCR results explored that most *B3* genes could be cold-induced and the expression is regulated by melatonin for the first time. We also found that *B3* genes could improve plant tolerance to cold stress through interaction with transcription factors, providing new insight into the role of the *B3* gene in soybean.

## Data availability statement

The data presented in the study are deposited in the NCBI repository, accession number PRJNA916852.

## Author contributions

CR: Methodology, Formal analysis, Writing – original draft. HW: Investigation. ZZ: Data curation. JJ: Software. QZ: Investigation. CL: Data curation. WL: Data curation. YZ: Resources, Funding acquisition. GY: Conceptualization, Writing – review & editing. All authors have read and agreed to the published version of the manuscript.
