# Supplementary material for: Genome-wide identification of the B3 gene family in soybean and the response to melatonin under cold stress
Source: Front Plant Sci. 2023 Jan 13;13:1091907. doi: 10.3389/fpls.2022.1091907 (PMC9880549; doi:10.3389/fpls.2022.1091907)
Supplement: Supplementary file 1 [file Table_1.doc]

**Table S1**

**Primers of *B3* genes in Figure 3**

| Primer name | Primer sequence |
| --- | --- |
| GmB3-001F | ATTTGCACGACACCGTTTGG |
| GmB3-001R | AGTTGTGGAGCGGTCTGAAG |
| GmB3-003F | ACCCAGCGAAACTCCAGATG |
| GmB3-003R | TCAGGTTTCGGTCGTGTCTG |
| GmB3-049F | TTCGGTCAGAATGAACGGCA |
| GmB3-049R | TACTGATTGCCCTGCACCAG |
| GmB3-078F | TCAGTCCTTGCTGACCGAAC |
| GmB3-078R | TCCCTGTCGACGAACACAAG |
| GmB3-083F | GTCTCTGGTGATGCAGTGCT |
| GmB3-083R | GGCAACCTGTAGACCTGTCC |
| GmB3-093F | CATGGCCAGGAATGGAGGTT |
| GmB3-093R | CTCCATCCTCACCCCTGAGA |
| GmB3-095F | AGCTTCAGACACCAGCACTC |
| GmB3-095R | AGTTGTGAGCAGATGCCTCC |
| GmB3-108F | CTCAGCCTGAAGGATTGCCA |
| GmB3-108R | CAAGCACTGGAGCTTCCTCA |
| GmB3-123F | TCCACTGCCAACAAACTGGT |
| GmB3-123R | TGAGCATGATTCCAACGGCT |

**Table S2**

**Physicochemical parameters of *GmB3* genes**

| Gene name | Gene_id | Chr | Location | protein length | CDS length | Isoelectric point | Molecular weight | instability index | aliphatic index |
| --- | --- | --- | --- | --- | --- | --- | --- | --- | --- |
| GmB3-01 | Glyma.01G002100 | 1 | 291328：297375 | 1104 | 3305 | 6.51 | 121957.83 | 63.38 | 76.1 |
| GmB3-02 | Glyma.01G087500 | 1 | 26423047：26424870 | 384 | 1155 | 9.25 | 41907.12 | 68.54 | 45.81 |
| GmB3-03 | Glyma.01G103500 | 1 | 34600880：34605669 | 692 | 2079 | 6.02 | 76961.62 | 70.36 | 46.67 |
| GmB3-04 | Glyma.01G133000 | 1 | 45250013：45258009 | 908 | 2727 | 6.41 | 101006.64 | 68.74 | 55.23 |
| GmB3-05 | Glyma.01G244800 | 1 | 56723159：56725021 | 361 | 1086 | 5.54 | 40818.78 | 32.24 | 94.57 |
| GmB3-06 | Glyma.02G099500 | 2 | 9306726：9308948 | 401 | 1206 | 9.16 | 43610.17 | 40.49 | 71.00 |
| GmB3-07 | Glyma.02G200500 | 2 | 38400034：38402766 | 344 | 1035 | 6.38 | 39079.69 | 59.56 | 50.41 |
| GmB3-08 | Glyma.02G236400 | 2 | 42391815：42399602 | 718 | 2157 | 6.49 | 80089.82 | 44.49 | 63.18 |
| GmB3-09 | Glyma.02G237500 | 2 | 42582609：42591402 | 407 | 1224 | 9.29 | 46060.16 | 51.06 | 68.8 |
| GmB3-10 | Glyma.02G239600 | 2 | 42813400：42824492 | 847 | 2544 | 6.06 | 94202.81 | 61.67 | 73.55 |
| GmB3-11 | Glyma.02G281700 | 2 | 46401584：46410932 | 896 | 2691 | 6.04 | 98952.51 | 67.86 | 75.31 |
| GmB3-12 | Glyma.02G291400 | 2 | 47082013：47082744 | 224 | 675 | 9.5 | 25804.63 | 37.68 | 72.23 |
| GmB3-13 | Glyma.03G035200 | 3 | 4265953：4274479 | 905 | 2718 | 6.34 | 100286.81 | 54.76 | 68.55 |
| GmB3-14 | Glyma.03G070500 | 3 | 16766136：16770934 | 691 | 2076 | 6.02 | 76833.4 | 47.27 | 68.67 |
| GmB3-15 | Glyma.03G199000 | 3 | 40799933：40802102 | 288 | 867 | 6.57 | 32721.87 | 64.66 | 49.76 |
| GmB3-16 | Glyma.03G208800 | 3 | 41564516：41575322 | 590 | 1773 | 6.9 | 65327.86 | 53.9 | 76.17 |
| GmB3-17 | Glyma.03G246400 | 3 | 44338409：44341213 | 308 | 927 | 9.19 | 35485.6 | 43.3 | 70.52 |
| GmB3-18 | Glyma.03G258300 | 3 | 45279918：45284681 | 662 | 1989 | 6.62 | 74073.74 | 58.93 | 70.2 |
| GmB3-19 | Glyma.03G262200 | 3 | 45556475：45559485 | 420 | 1263 | 6.79 | 47128.11 | 48.31 | 64.1 |
| GmB3-20 | Glyma.04G037800 | 4 | 3007134：3012360 | 228 | 687 | 9.52 | 25763.56 | 57.13 | 85.53 |
| GmB3-21 | Glyma.04G037900 | 4 | 3013602：3018148 | 185 | 558 | 5.15 | 21100.06 | 28.9 | 83.24 |
| GmB3-22 | Glyma.04G133200 | 4 | 18932507：18933889 | 186 | 561 | 7.72 | 21903.95 | 39.6 | 76.45 |
| GmB3-23 | Glyma.04G143300 | 4 | 26120011：26120532 | 173 | 522 | 9.3 | 20205.13 | 55.16 | 67.05 |
| GmB3-24 | Glyma.04G156400 | 4 | 36758125：36758770 | 173 | 522 | 9.35 | 20123.05 | 53.48 | 67.05 |
| GmB3-25 | Glyma.04G192400 | 4 | 46416413：46421655 | 613 | 1842 | 5.93 | 69014.87 | 48.07 | 70.73 |
| GmB3-26 | Glyma.04G200600 | 4 | 47316151：47321158 | 843 | 2532 | 6.3 | 93884.53 | 56.04 | 65.47 |
| GmB3-27 | Glyma.04G254200 | 4 | 52092830：52097184 | 562 | 1689 | 5.53 | 61209.95 | 48.05 | 71.46 |
| GmB3-28 | Glyma.04G257000 | 4 | 52314502：52328177 | 812 | 2439 | 9.63 | 91347.7 | 46.84 | 63.87 |
| GmB3-29 | Glyma.05G143800 | 5 | 33756023：33763613 | 866 | 2601 | 6.13 | 95857.36 | 67.65 | 70.57 |
| GmB3-30 | Glyma.05G200800 | 5 | 38463201：38468505 | 858 | 2577 | 6.35 | 94845.13 | 52.13 | 67.02 |
| GmB3-31 | Glyma.05G221300 | 5 | 40073989：40080807 | 1099 | 3300 | 6.01 | 120828.59 | 61.52 | 80.35 |
| GmB3-32 | Glyma.06G038900 | 6 | 2943383：2947586 | 232 | 699 | 9.30 | 26278.29 | 65.19 | 87.84 |
| GmB3-33 | Glyma.06G164900 | 6 | 13670852：13675929 | 843 | 2532 | 6.19 | 93667.2 | 59.4 | 63.49 |
| GmB3-34 | Glyma.06G207800 | 6 | 20207077：20207940 | 234 | 705 | 9.74 | 27128.15 | 57.73 | 65.81 |
| GmB3-35 | Glyma.06G213700 | 6 | 21727968：21730998 | 138 | 417 | 6.27 | 16187.76 | 52.24 | 87.54 |
| GmB3-36 | Glyma.06G220000 | 6 | 25283455：25285827 | 306 | 921 | 9.78 | 35509.9 | 38.72 | 67.55 |
| GmB3-37 | Glyma.06G223000 | 6 | 29303903：29304917 | 243 | 732 | 7.73 | 28413.96 | 35.89 | 94.16 |
| GmB3-38 | Glyma.06G228300 | 6 | 34673427：34674329 | 142 | 429 | 7.82 | 16960.24 | 29.69 | 74.01 |
| GmB3-39 | Glyma.07G048200 | 7 | 4061864：4064513 | 500 | 1503 | 6.6 | 56285.55 | 55.34 | 54.84 |
| GmB3-40 | Glyma.07G054800 | 7 | 4807176：4812376 | 716 | 2151 | 6.07 | 79530.35 | 52.65 | 70.8 |
| GmB3-41 | Glyma.07G130400 | 7 | 15465920：15471704 | 1110 | 3333 | 6.3 | 122978.03 | 63.79 | 75.15 |
| GmB3-42 | Glyma.07G134800 | 7 | 15999948：16005619 | 664 | 1995 | 5.99 | 74516.94 | 51.99 | 74.23 |
| GmB3-43 | Glyma.07G157300 | 7 | 19363580：19365227 | 322 | 969 | 8.4 | 37687.67 | 41.68 | 68.01 |
| GmB3-44 | Glyma.07G163700 | 7 | 21690647：21696235 | 468 | 1407 | 8.75 | 53047.07 | 39.46 | 75.79 |
| GmB3-45 | Glyma.07G202200 | 7 | 37125021：37130940 | 709 | 2130 | 6.67 | 77934.52 | 52.83 | 73.27 |
| GmB3-46 | Glyma.07G224100 | 7 | 40138227：40141705 | 128 | 549 | 7.74 | 20427.64 | 47.06 | 92.03 |
| GmB3-47 | Glyma.07G272800 | 7 | 44495749：44503862 | 674 | 2025 | 5.92 | 75123.46 | 57.8 | 71.99 |
| GmB3-48 | Glyma.08G008100 | 8 | 643138：649093 | 851 | 2556 | 6.39 | 94421.63 | 55.03 | 68.51 |
| GmB3-49 | Glyma.08G027800 | 8 | 2221005：2228300 | 1113 | 3342 | 5.86 | 122551.22 | 63.04 | 77.85 |
| GmB3-50 | Glyma.08G100100 | 8 | 7673674：7680574 | 905 | 2718 | 6.17 | 100354.68 | 65.04 | 72.48 |
| GmB3-51 | Glyma.08G327600 | 8 | 44568950：44569420 | 156 | 471 | 9.07 | 18735.45 | 44.51 | 87.37 |
| GmB3-52 | Glyma.08G327800 | 8 | 44575624：44576094 | 156 | 471 | 8.78 | 18696.37 | 42.48 | 85.51 |
| GmB3-53 | Glyma.08G339100 | 8 | 45552867：45554694 | 256 | 771 | 9.1 | 29316.4 | 37.58 | 73.44 |
| GmB3-54 | Glyma.08G339200 | 8 | 45555910：45557944 | 285 | 858 | 9.59 | 32870.2 | 45.98 | 66.00 |
| GmB3-55 | Glyma.08G357600 | 8 | 47001120：47005697 | 761 | 2286 | 5.52 | 83848.44 | 46.97 | 55.02 |
| GmB3-56 | Glyma.09G072200 | 9 | 7478279：7486860 | 1125 | 3378 | 6.02 | 125840.87 | 67.64 | 67.01 |
| GmB3-57 | Glyma.09G114900 | 9 | 24804219：24807190 | 360 | 1083 | 7.26 | 40776.35 | 40.25 | 73.36 |
| GmB3-58 | Glyma.09G117200 | 9 | 27377355：27380798 | 528 | 1157 | 9.19 | 60548.44 | 40.63 | 79.91 |
| GmB3-59 | Glyma.09G117500 | 9 | 27590976：27594675 | 621 | 1866 | 6.15 | 70560 | 51.87 | 76.63 |
| GmB3-60 | Glyma.09G194300 | 9 | 41867600：41868959 | 177 | 534 | 5.69 | 20401.02 | 30.95 | 83.62 |
| GmB3-61 | Glyma.10G053500 | 10 | 4838661：4843964 | 700 | 2103 | 7.26 | 77266.44 | 46.42 | 73.39 |
| GmB3-62 | Glyma.10G076100 | 10 | 7918148：7921127 | 337 | 1014 | 6.46 | 38494.02 | 58.23 | 46.26 |
| GmB3-63 | Glyma.10G103500 | 10 | 21855557：21856051 | 164 | 495 | 9.26 | 18570.4 | 53.16 | 75.98 |
| GmB3-64 | Glyma.10G204400 | 10 | 43557479：43559329 | 351 | 1056 | 8.07 | 38379.29 | 37.31 | 69.97 |
| GmB3-65 | Glyma.10G210600 | 10 | 44284423：44287975 | 612 | 1839 | 7.21 | 67127.69 | 45.48 | 67.70 |
| GmB3-66 | Glyma.10G260300 | 10 | 48585388：48585782 | 95 | 288 | 11.02 | 11210.08 | 23.46 | 72.84 |
| GmB3-67 | Glyma.10G281100 | 10 | 50240906：50243956 | 387 | 1161 | 7.44 | 45727.9 | 39.85 | 72.25 |
| GmB3-68 | Glyma.10G281200 | 10 | 50246999：50249746 | 367 | 1104 | 9.62 | 42586.04 | 43.64 | 78.56 |
| GmB3-69 | Glyma.10G281300 | 10 | 50251157：50252837 | 316 | 951 | 9.08 | 36871.19 | 51.32 | 65.32 |
| GmB3-70 | Glyma.11G124000 | 11 | 9423928：9432266 | 242 | 729 | 4.9 | 28476.23 | 70.64 | 55.99 |
| GmB3-71 | Glyma.11G124100 | 11 | 9434184：9437964 | 431 | 1296 | 9 | 48890.56 | 51.32 | 74.83 |
| GmB3-72 | Glyma.11G124200 | 11 | 9440642：9444013 | 434 | 1305 | 9.36 | 50059.36 | 49.85 | 62.28 |
| GmB3-73 | Glyma.11G124300 | 11 | 9449136：9451421 | 337 | 1014 | 7.57 | 38771.2 | 40.81 | 74.3 |
| GmB3-74 | Glyma.11G125200 | 11 | 9511103：9514048 | 336 | 1011 | 5.13 | 39472.75 | 57.25 | 59.46 |
| GmB3-75 | Glyma.11G145500 | 11 | 11195651：11199400 | 697 | 2094 | 7.85 | 76555.96 | 43.9 | 74.42 |
| GmB3-76 | Glyma.11G197900 | 11 | 27469452：27478048 | 911 | 2736 | 7.28 | 100738.66 | 42.68 | 63.15 |
| GmB3-77 | Glyma.11G198800 | 11 | 27617995：27618919 | 105 | 318 | 9.71 | 12069.99 | 31.78 | 66.86 |
| GmB3-78 | Glyma.11G204200 | 11 | 28422034：28429861 | 844 | 2535 | 5.93 | 93600.09 | 58.73 | 75.89 |
| GmB3-79 | Glyma.12G048500 | 12 | 3499773：3502868 | 310 | 933 | 6.64 | 36350.23 | 53.53 | 72.94 |
| GmB3-80 | Glyma.12G048600 | 12 | 3504850：3508614 | 441 | 1326 | 9.19 | 50118.12 | 51.78 | 77.8 |
| GmB3-81 | Glyma.12G048700 | 12 | 3512183：3514061 | 315 | 948 | 7.60 | 36667.95 | 46.51 | 76.76 |
| GmB3-82 | Glyma.12G049900 | 12 | 3577879：3579075 | 286 | 861 | 6.09 | 33486.86 | 56.99 | 77.03 |
| GmB3-83 | Glyma.12G071000 | 12 | 5197212：5204646 | 792 | 2379 | 6.00 | 87731.72 | 53.18 | 73.83 |
| GmB3-84 | Glyma.12G076200 | 12 | 5851956：5855787 | 701 | 2106 | 8.24 | 77188.67 | 43.03 | 73.59 |
| GmB3-85 | Glyma.12G119700 | 12 | 12727693：12730092 | 112 | 339 | 9.72 | 13215.34 | 51.58 | 96.61 |
| GmB3-86 | Glyma.12G164100 | 12 | 31863013：31869597 | 665 | 1998 | 5.75 | 74273.52 | 60.82 | 67.98 |
| GmB3-87 | Glyma.12G171000 | 12 | 32643495：32650065 | 799 | 2400 | 6.49 | 88732.31 | 55.71 | 70.53 |
| GmB3-88 | Glyma.12G174100 | 12 | 33132698：33136308 | 700 | 2103 | 7.60 | 76824.93 | 48.61 | 75.07 |
| GmB3-89 | Glyma.13G058500 | 13 | 15593710：15595584 | 306 | 921 | 8.49 | 35089.77 | 38.06 | 70.33 |
| GmB3-90 | Glyma.13G084700 | 13 | 19586384：19590900 | 551 | 1656 | 5.78 | 60677.92 | 52.46 | 63.36 |
| GmB3-91 | Glyma.13G112600 | 13 | 22585808：22593464 | 1131 | 3396 | 6.08 | 126747.12 | 65.28 | 73.47 |
| GmB3-92 | Glyma.13G140600 | 13 | 25308345：25312385 | 514 | 1545 | 8.93 | 57052.25 | 45.45 | 74.94 |
| GmB3-93 | Glyma.13G174000 | 13 | 28805502：28811556 | 714 | 2145 | 6.67 | 78286.47 | 54.87 | 70.31 |
| GmB3-94 | Glyma.13G221400 | 13 | 33457802：33464856 | 896 | 2961 | 6.28 | 99837.22 | 60.45 | 71.91 |
| GmB3-95 | Glyma.13G234200 | 13 | 34492285：34498651 | 736 | 2211 | 6.25 | 80446.53 | 56.35 | 74.97 |
| GmB3-96 | Glyma.13G245900 | 13 | 35481859：35492023 | 879 | 2640 | 7.4 | 96869.31 | 55.87 | 67.84 |
| GmB3-97 | Glyma.13G325200 | 13 | 42036714：42039907 | 670 | 2013 | 7.61 | 73760.45 | 48.92 | 75.99 |
| GmB3-98 | Glyma.13G328000 | 13 | 42266563：42271620 | 620 | 1863 | 6.06 | 68962.74 | 51.55 | 70.45 |
| GmB3-99 | Glyma.14G032700 | 14 | 2372881：2381492 | 896 | 2691 | 6.32 | 98724.15 | 65.26 | 75.32 |
| GmB3-100 | Glyma.14G055700 | 14 | 4424574：4426279 | 317 | 954 | 4.86 | 37113.31 | 48.68 | 67.6 |
| GmB3-101 | Glyma.14G079100 | 14 | 6762702：6766585 | 455 | 1338 | 8.3 | 49788 | 44.19 | 66.34 |
| GmB3-102 | Glyma.14G079200 | 14 | 6774233：6778714 | 600 | 1803 | 8.68 | 67298.63 | 36.04 | 74.03 |
| GmB3-103 | Glyma.14G158000 | 14 | 34985140：34995244 | 215 | 648 | 9.75 | 25744.5 | 43.87 | 65.67 |
| GmB3-104 | Glyma.14G166500 | 14 | 41161768：41166035 | 519 | 1560 | 6.74 | 57239.25 | 54.44 | 63.89 |
| GmB3-105 | Glyma.14G204400 | 14 | 46936057：46943868 | 724 | 2175 | 7.52 | 80490.53 | 44.72 | 66.15 |
| GmB3-106 | Glyma.14G208500 | 14 | 47412802：47421148 | 843 | 2532 | 6.01 | 93795.44 | 60.63 | 74.12 |
| GmB3-107 | Glyma.14G217700 | 14 | 48242771：48249860 | 930 | 2793 | 5.62 | 103499.15 | 53.23 | 78.17 |
| GmB3-108 | Glyma.15G067900 | 15 | 5180434：5191496 | 878 | 2637 | 7.83 | 96724.13 | 55.83 | 66.91 |
| GmB3-109 | Glyma.15G078800 | 15 | 6049763：6053996 | 720 | 2163 | 6.06 | 78858.49 | 53.61 | 74.47 |
| GmB3-110 | Glyma.15G091000 | 15 | 6983384：6990249 | 898 | 2697 | 6.22 | 99689.89 | 57.02 | 73.82 |
| GmB3-111 | Glyma.15G181000 | 15 | 17479272：17487578 | 1122 | 3369 | 6.19 | 125284.27 | 67.09 | 67.9 |
| GmB3-112 | Glyma.16G000300 | 16 | 25179：31940 | 665 | 1998 | 5.62 | 74193.35 | 58.61 | 70.47 |
| GmB3-113 | Glyma.16G017100 | 16 | 1483694：1486538 | 582 | 1749 | 6.96 | 65550.71 | 62.17 | 55.64 |
| GmB3-114 | Glyma.16G023600 | 16 | 2253326：2258523 | 716 | 2151 | 6.06 | 79453.58 | 53.4 | 72.84 |
| GmB3-115 | Glyma.16G046700 | 16 | 4439905：4444017 | 363 | 1092 | 9.75 | 41567.09 | 34.24 | 78.37 |
| GmB3-116 | Glyma.16G050300 | 16 | 4817240：4821114 | 338 | 1017 | 6.35 | 38074.48 | 45.5 | 71.48 |
| GmB3-117 | Glyma.17G047100 | 17 | 3560557：3568575 | 1136 | 3411 | 6.05 | 127058.28 | 67.23 | 72.9 |
| GmB3-118 | Glyma.17G175500 | 17 | 18335035：18339356 | 480 | 1443 | 6.88 | 54328.76 | 47.93 | 77.4 |
| GmB3-119 | Glyma.17G245900 | 17 | 40134685：40138617 | 562 | 1689 | 8.73 | 63251.27 | 34.29 | 74.89 |
| GmB3-120 | Glyma.17G246000 | 17 | 40142108：40147051 | 439 | 1320 | 8.63 | 48991.37 | 48.04 | 68.11 |
| GmB3-121 | Glyma.17G256500 | 17 | 41032305：41037627 | 933 | 2802 | 5.48 | 103492.16 | 51.5 | 77.19 |
| GmB3-122 | Glyma.18G046800 | 18 | 4089351：4096885 | 841 | 2526 | 5.93 | 93247.82 | 57.98 | 76.05 |
| GmB3-123 | Glyma.18G052100 | 18 | 4503897：4512049 | 935 | 2808 | 7.44 | 103149.89 | 42.97 | 64.03 |
| GmB3-124 | Glyma.18G079800 | 18 | 7649539：7649961 | 116 | 351 | 9.6 | 13528.58 | 44.56 | 93.19 |
| GmB3-125 | Glyma.18G155000 | 18 | 32216208：32218820 | 344 | 1035 | 10.04 | 39761.34 | 43.03 | 77.38 |
| GmB3-126 | Glyma.18G176100 | 18 | 41894037：41898552 | 758 | 2277 | 5.72 | 83607.43 | 51.14 | 56.25 |
| GmB3-127 | Glyma.18G184500 | 18 | 44451085：44457655 | 664 | 1995 | 5.89 | 74553.01 | 51.79 | 76.46 |
| GmB3-128 | Glyma.19G100900 | 19 | 34806060：34810057 | 332 | 999 | 6.51 | 37400.86 | 44.87 | 70.72 |
| GmB3-129 | Glyma.19G105300 | 19 | 35505238：35507021 | 250 | 753 | 9.98 | 29180.56 | 39.59 | 74.84 |
| GmB3-130 | Glyma.19G181900 | 19 | 44046183：44050281 | 700 | 2103 | 8.3 | 77788.51 | 50.38 | 71.2 |
| GmB3-131 | Glyma.19G196600 | 19 | 45373108：45375062 | 299 | 900 | 6.48 | 34108.18 | 60.08 | 49.26 |
| GmB3-132 | Glyma.19G206100 | 19 | 46167548：46178826 | 677 | 2034 | 7.11 | 75645.75 | 57.2 | 73.87 |
| GmB3-133 | Glyma.19G261300 | 19 | 50457092：50462814 | 413 | 1242 | 7.49 | 45902.94 | 47.34 | 66.15 |
| GmB3-134 | Glyma.20G008700 | 20 | 772084：777840 | 435 | 1308 | 8.96 | 49452.85 | 44.33 | 73.91 |
| GmB3-135 | Glyma.20G035700 | 20 | 5007624：5010250 | 377 | 1134 | 5.97 | 42642.63 | 47.54 | 68.01 |
| GmB3-136 | Glyma.20G035800 | 20 | 5023078：5025544 | 377 | 1134 | 6.28 | 42681.66 | 43.75 | 69.28 |
| GmB3-137 | Glyma.20G107900 | 20 | 35044042：35046452 | 430 | 1293 | 8.81 | 49756.83 | 40.24 | 72.47 |
| GmB3-138 | Glyma.20G108000 | 20 | 35047833：35051623 | 454 | 1365 | 9.05 | 53298.04 | 51.18 | 70.79 |
| GmB3-139 | Glyma.20G108100 | 20 | 35057879：35058655 | 128 | 387 | 8.95 | 14725.78 | 50.98 | 76.88 |
| GmB3-140 | Glyma.20G108200 | 20 | 35059793：35061991 | 72 | 219 | 8.95 | 8632.16 | 41.01 | 82.5 |
| GmB3-141 | Glyma.20G108300 | 20 | 35066655：35068285 | 298 | 897 | 9.31 | 34433.72 | 40.78 | 82.99 |
| GmB3-142 | Glyma.20G108400 | 20 | 35076210：35077822 | 267 | 804 | 6.26 | 30895.34 | 33.83 | 84.68 |
| GmB3-143 | Glyma.20G180000 | 20 | 41780308：41783535 | 593 | 1782 | 6.7 | 65246.96 | 46.04 | 70.2 |
| GmB3-144 | Glyma.20G186200 | 20 | 42482796：42483988 | 362 | 1089 | 9.08 | 39343.56 | 35.8 | 72.49 |
| GmB3-145 | Glyma.20G247300 | 20 | 47686501：47687460 | 319 | 960 | 8.08 | 36201.63 | 38.92 | 64.23 |
